# *Saccharomyces* yeast postbiotics mitigate mucosal damages from F18^+^ *Escherichia coli* challenges by positively balancing the mucosal microbiota in the jejunum of young pigs

**DOI:** 10.1186/s42523-024-00363-y

**Published:** 2024-12-20

**Authors:** Alexa R. Gormley, Marcos Elias Duarte, Zixiao Deng, Sung Woo Kim

**Affiliations:** Department of Animal Science, North Carolina State University, 116 Polk Hall, 120 W Broughton Dr, Raleigh, NC 27695 USA

**Keywords:** F18 + *Escherichia coli*, Mucosal immunity, Mucosal microbiota, Nursery pigs, *Saccharomyces* postbiotics

## Abstract

**Background:**

Enterotoxigenic *Escherichia coli* (*E. coli*) is one of the most prevalent causes of diarrhea in young animals. Postbiotics derived from yeast have the potential to positively influence the mucosal microbiota in the jejunum, therefore it was hypothesized that *Saccharomyces* yeast postbiotics could enhance the microbiota and mucosal immune response in the jejunum, mitigating the effects of infection with enterotoxigenic *E. coli*. The purpose of this study was to investigate the effects of a *Saccharomyces* yeast postbiotic on the mucosal microbiota and mucosal immune response in the jejunum of newly weaned pigs challenged with F18^+^ *E. coli.*

**Results:**

Thirty-six individually housed nursery pigs were allotted into three treatments utilizing a randomized complete block design; negative control (NC: basal diet, no challenge), positive control (PC: basal diet, challenge), and SYP (basal diet + *Saccharomyces* yeast postbiotics at 175 g/ton*,* challenge). On d 7, PC and SYP were orally inoculated with F18^+^ *E. coli*, whereas NC received saline. On d 28, pigs were euthanized for sampling of the jejunum to analyze the mucosal microbiota, oxidative stress, immune status, and intestinal morphology. The PC reduced (*P* < 0.05) growth performance compared to NC. The SYP improved (*P* < 0.05) fecal score from d 7–18 when compared with PC. SYP reduced (*P* < 0.05) protein carbonyl, reduced (*P* < 0.05) gene expression of Toll-like receptor 4, and increased (*P* < 0.05) gene expression of mammalian target of rapamycin, compared with PC.

**Conclusions:**

Challenge with F18^+^ *E. coli* negatively impacted jejunal mucosa-associated microbiota and jejunal morphology, affecting growth performance. *Saccharomyces* yeast postbiotics could reduce the negative effects associated with F18^+^ *E. coli* infection.

## Background

The mammalian intestine is critically important in maintaining overall health and well-being, specifically in young animals, including human infants [1] and young animals upon weaning [2]. The symbiotic relationship between intestinal epithelial cells and the microbial communities associated with the intestinal mucosa can determine the efficacy of nutrient absorption and bolster the intestinal immune response [3]. Enteric pathogens can cause or take advantage of disruptions to the typical populations of the mucosa-associated microbiota [4]. Enterotoxigenic *Escherichia coli* (ETEC) is a common enteric pathogen affecting humans and animals worldwide [5, 6]. Specifically, ETEC contributes to overall morbidity and mortality, especially in the young [7–9].

Common enterotoxins associated with ETEC can induce an electrolyte imbalance in the lumen of the jejunum, altering the environment and thereby influencing the mucosa-associated microbiota in the jejunum [10]. Historically, antibiotics have been utilized to mitigate the severity of a disease caused by certain pathogens, however, public concerns surrounding antimicrobial resistance has prompted researchers to investigate alternative solutions [11–13]. The use of postbiotics could contribute to the proliferation of beneficial microbial populations in the intestines, encouraging a positive interaction with the host by enhancing mucosal immune response [14–16]. Postbiotics are most commonly derived from yeast or bacteria, and the mechanism of action for the benefits associated with postbiotics is unique to the microorganism and the specific fragment or metabolite of the microorganism being utilized [17, 18]. Currently, the efficacy of bacterial and yeast postbiotics in mitigating the symptoms and intestinal damage associated with diarrhea is still being investigated as it relates to human applications, and bacterial postbiotics are often at the forefront of the postbiotic research [19–23].

In contrast, *Saccharomyces* spp*.* have been researched for their postbiotic and immunomodulatory properties with success in improving intestinal immune response being displayed in monogastric animals [24–27], indicating potential applications related to human health. It has been reported that *Saccharomyces* cell walls contain immunoregulatory properties that could prevent adherence of pathogenic bacteria to the intestine and promote the release of cytokines from macrophages, in addition to promoting proliferation of beneficial microbial populations in the intestines [28, 29]. In general, yeast cell walls are thought to be effective as postbiotics due to its structural components, including mannan oligosaccharides and β-D-glucans [30]. Specifically, mannan oligosaccharides have been shown to affect the intestinal immune response by enhancing immunocompetence and inducing an advantageous change of the host microbiota [31]. β-D-glucans have been shown to decrease pro-inflammatory cytokines, increase anti-inflammatory cytokines, decrease the population of pathogenic bacteria in the ileum and colon, and improve intestinal morphology parameters, thereby improving overall growth performance, when fed to pigs [32–34].

Due to the anatomical and physiological organization of the porcine intestinal tract, pigs serve as an appropriate model to investigate the effects of various nutritional interventions on enteric disease and dysfunction. As such, nursery pigs were utilized to serve as a research model for young monogastric animals and infants, experiencing diarrhea as a result of infection with ETEC. Specifically, ETEC colonizes the small intestine by adhering to enterocytes using a strain-specific fimbriae or pili, and the F18^+^ *E. coli* strain has been identified as one of the most prevalent strain causing diarrhea in nursery pigs [35–37].

Therefore, it was hypothesized that *Saccharomyces* yeast postbiotics could increase the populations of beneficial microorganisms in the jejunal mucosa and enhance the mucosal immune response, thereby decreasing intestinal inflammation and oxidative stress related to infection with pathogenic F18^+^ *E. coli* in newly weaned pigs. Thus, the objective of this study was to investigate the effects of a *Saccharomyces* yeast postbiotic on the mucosal microbiota and mucosal immune response in the jejunum of newly weaned pigs challenged with F18^+^ *E. coli.*

## Results

### Profiling of the jejunal mucosa-associated microbiota

At the genus level, there was no difference in α-diversity measures observed between the NC and PC groups, whereas SYP decreased (*P* < 0.05) the Chao1 α-diversity when compared to the PC group (Table 1).

**Table 1:** Alpha diversity of mucosa-associated microbiota in the jejunum of nursery pigs fed diets supplemented with *Saccharomyces* yeast postbiotic

Item	Treatment^a^				*P* value	
	NC	PC	SYP	SEM	NC versus PC	PC versus SYP
Chao1	46.6	49.3	41.6	2.9	0.375	0.018
Shannon	1.57	1.75	1.75	0.20	0.530	0.991
Simpson	0.64	0.68	0.70	0.07	0.673	0.546

PC, NC + challenged with F18^+^ *E. coli*; SYP, NC + *Saccharomyces* yeast postbiotics (175 g/ton of feed) + challenged with F18^+^ *E. coli*

^a^NC, basal diet (no supplementation)

**Fig. 1:**
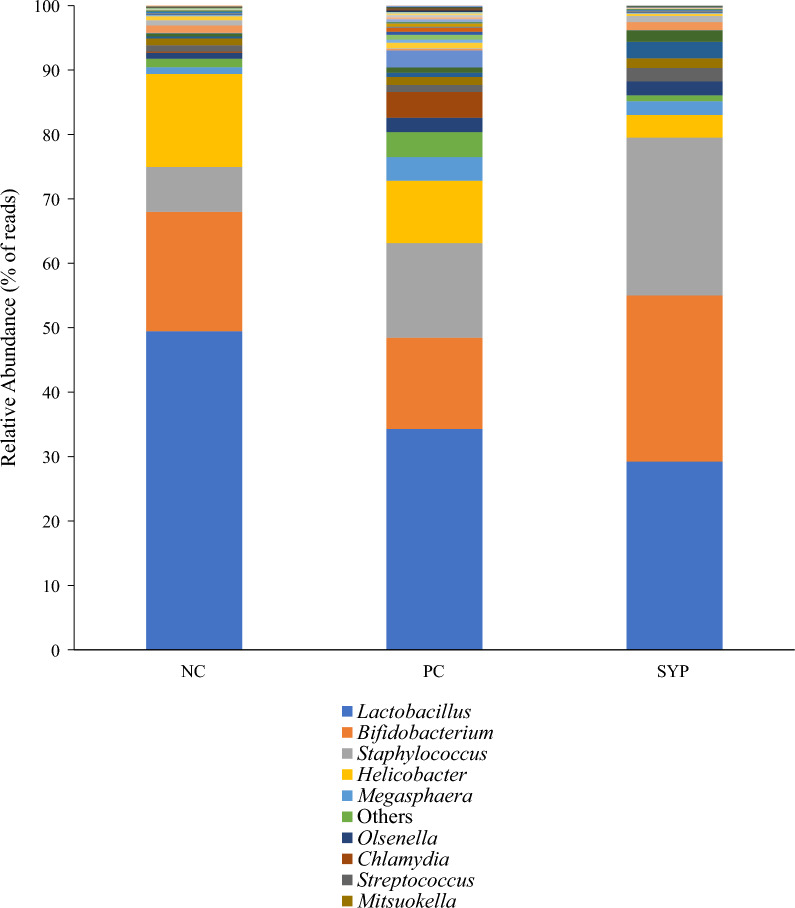
Relative abundance of the jejunal mucosa-associated microbiota at the genus level in nursery pigs fed diets supplements with a *Saccharomyces* yeast postbiotic. A genus was considered a part of “Others” when the relative abundance was < 0.5%. The top 10 most abundant genus across the treatments are identified in the legend

**Fig. 2:**
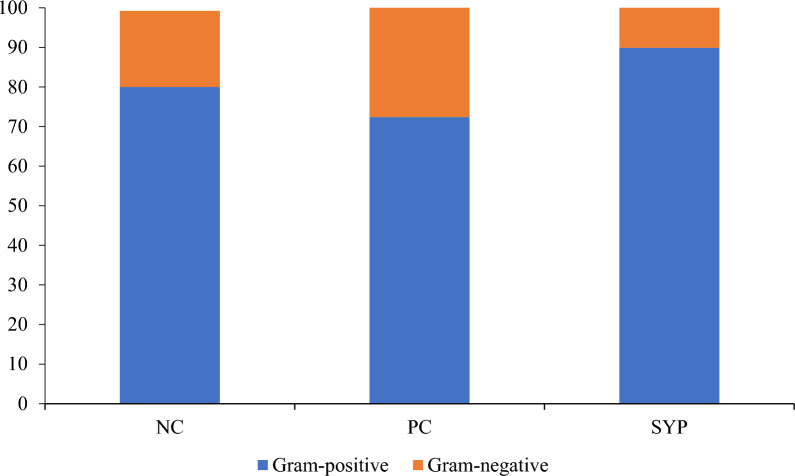
Relative abundance of the jejunal mucosa-associated microbiota at the genus level in nursery pigs fed diets supplements with a *Saccharomyces* yeast postbiotic, grouped by proportion of gram-positive to gram-negative bacteria. The proportion of gram-positive to gram-negative bacteria was determined by removing “Others” and identifying the abundance of gram-positive and -negative bacteria from the known species. A genus was considered a part of “Others” when the relative abundance was < 0.5%

**Fig. 3:**
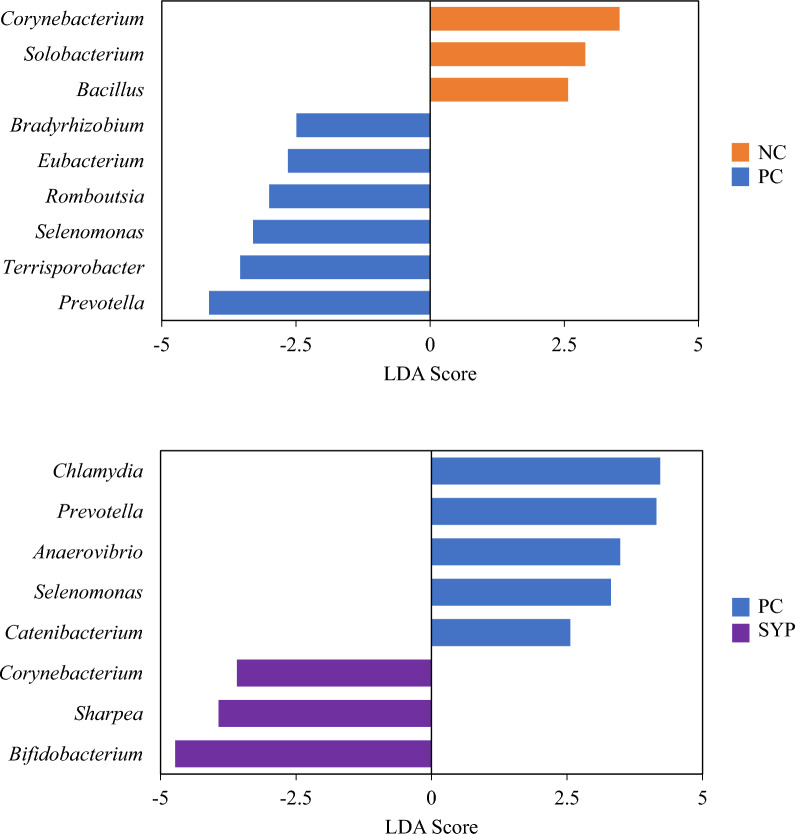
Linear discriminant analysis (LDA) effect size (LEfSe) of differentially abundant genera between the NC and PC, and PC and SYP treatment groups, respectively

**Fig. 4:**
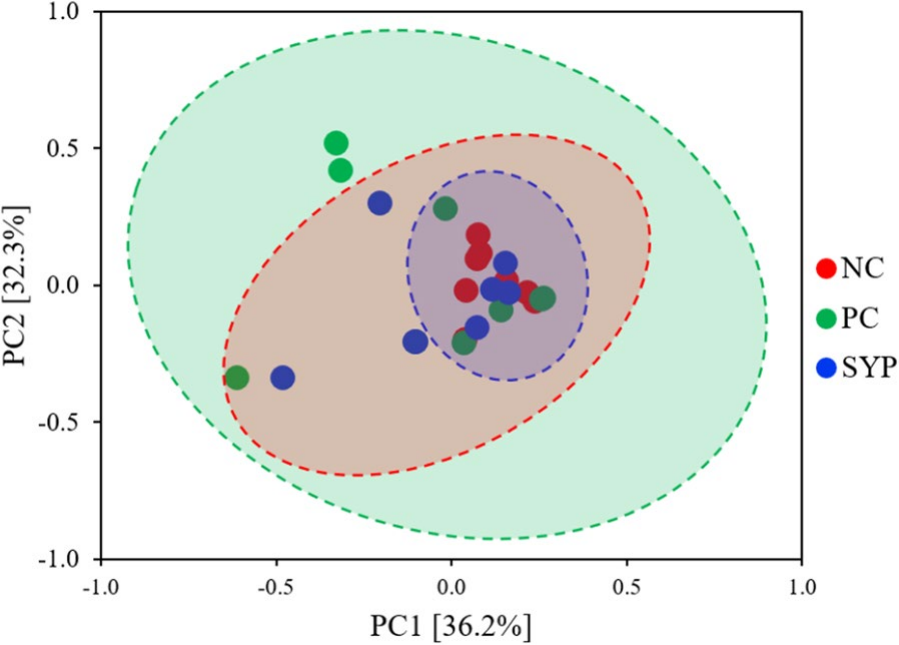
Principal component analysis (PCoA) plot in the jejunal mucosa-associated microbiota at the genus level in nursery pigs fed diets supplemented with a *Saccharomyces* yeast postbiotic. The X-axis and Y-axis represent the principal component axes, with the percentages indicating the proportion of variation explained by each component. Points of different colors correspond to samples from different treatments (NC, PC, and SYP), and the close two points are, the more similar the points are in their species composition. The *P-*value for the Bray–Curtis between NC and PC was 0.608 and between PC and SYP was 0.292

In all treatments, the genera *Lactobacillus, Bifidobacterium,* and *Staphylococcus* were the three most abundant by number of total reads (Fig. 1). The PC group (27.61%) had a greater percentage of gram-negative bacteria when compared with the NC group (19.19%), whereas the SYP group (10.08%) had the lowest relative abundance of gram-negative bacteria across the treatments (Fig. 2). The LEfSe analysis identified that the PC group increased the relative abundance of *Bradyrhizobium, Eubacterium, Rombotsia, Selemonas, Terrisporobacter,* and *Prevotella* and decreased the relative abundance of *Corynebacterium, Solobacterium,* and *Bacillus* when compared with the NC group (Fig. 3). In addition, the LEfSe analysis identified that the SYP group increased the relative abundance of *Corynebacterium, Sharpea,* and *Bifidobacterium* and decreased the relative abundance of *Chlamydia, Prevotella, Anaerovibrio, Selenomonas,* and *Catenibacterium* when compared with the PC group. The microbial communities at the genus level were visualized using PCoA based on Bray–Curtis distance and no differences were found across the treatments in regards to beta diversity (Fig. 4).

### Relative mRNA expression of genes in jejunal tissue

In jejunal tissue, the relative gene expression of Toll-like receptor (TLR) 2 was unchanged when comparing NC and PC, as well as when comparing PC and SYP (Table 2). In contrast, the relative gene expression of TLR4 was increased (*P* < 0.05) in the PC when compared with the NC, whereas it was decreased (*P* < 0.05) in SYP when compared with the PC. The relative gene expression of nucleotide binding oligomerization domain containing (NOD) 1 in jejunal tissue was unaffected between NC and PC, and between PC and SYP. Similarly, relative gene expression of NOD2 did not differ between NC and PC or the SYP and PC. Interestingly, the relative expression of mammalian target of rapamycin (mTOR) was unaffected when comparing the NC and PC groups, however, relative expression of mTOR increased (*P* < 0.05) in SYP when compared to the PC. The relative gene expression of claudin showed no difference between the NC and PC, as well as no difference between the SYP and PC. The relative gene expression of occludin tended to be decreased (*P* = 0.061) in the NC when compared to the PC, whereas it was not affected when comparing SYP and PC. Finally, the relative gene expression of zonula occludens-1 were also unaffected when comparing NC and PC, as well as when comparing SYP and PC.

**Table 2:** Relative gene expression in jejunal tissue of nursery pigs fed diets supplemented with *Saccharomyces* yeast postbiotics

Item	Treatment^a^				*P* value	
	NC	PC	SYP	SEM	NC versus PC	PC versus SYP
Toll-like receptor 2	1.12	1.00	1.07	0.20	0.684	0.819
Toll-like receptor 4	0.99	3.00	0.86	0.62	0.029	0.021
NOD^b^1	1.03	1.02	1.17	0.15	0.964	0.511
NOD2	1.05	1.11	1.03	0.12	0.746	0.529
Mammalian target of rapamycin	1.18	1.00	1.32	0.13	0.278	0.035
Claudin	1.97	1.97	2.34	0.75	0.996	0.735
Occludin	1.02	1.26	1.24	0.09	0.061	0.836
Zonula occludens-1	1.01	1.00	1.01	0.07	0.892	0.963

^a^NC, basal diet (no supplementation); PC, NC + challenged with F18^+^ *E. coli*; SYP, NC + *Saccharomyces* yeast postbiotics (175 g/ton of feed) + challenged with F18^+^ *E. coli*

^b^NOD, Nucleotide binding oligomerization domain containing

### Oxidative stress status, humoral immune status, and intestinal inflammatory status

Malondialdehyde (MDA) concentration in the jejunal mucosa was not affected by PC or SYP (Table 3). Protein carbonyl concentration in the jejunal mucosa was not affected between the PC and NC, however, the concentration was decreased (*P* < 0.05) by SYP when compared with PC. The concentrations of the immunoglobulins (IgG and IgA) in the jejunal mucosa were not affected in PC when compared to NC, nor where they affected when comparing SYP with PC. The concentrations of the pro-inflammatory cytokine TNF-α and IL-8 were unaffected when comparing NC to PC and were also unaffected when comparing SYP with PC. In contrast, the concentrations of the pro-inflammatory cytokine IL-6 tended to be reduced (*P* = 0.073) in PC when compared with the NC, whereas it was not affected by SYP.

**Table 3:** Products of oxidative damage and markers of immune status in nursery pigs fed diets supplemented with *Saccharomyces* yeast postbiotic

Item	Treatment^a^				*P* value	
	NC	PC	SYP	SEM	NC versus PC	PC versus SYP
Unit/mg protein						
MDA, nmol	0.34	0.46	0.48	0.07	0.205	0.874
Protein carbonyl, nmol	1.28	1.59	0.73	0.27	0.332	0.011
IgG, µg	1.85	1.95	1.82	0.30	0.806	0.742
IgA, µg	2.76	2.84	3.17	0.42	0.917	0.582
TNFa, pg	4.15	3.78	4.60	0.91	0.736	0.451
IL-6, pg	39.12	22.22	37.23	6.56	0.073	0.109
IL-8, pg	1.31	1.46	1.43	0.20	0.592	0.900

PC, NC + challenged with F18^+^ *E. coli*; SYP, NC + *Saccharomyces* yeast postbiotics (175 g/ton of feed) + challenged with F18^+^ *E. coli*

^a^NC, basal diet (no supplementation)

### Intestinal morphology and crypt cell proliferation

Villus height tended to be reduced (*P* = 0.041) in the PC group when compared with NC, whereas the villus height of pigs in SYP were unaffected when compared with PC (Table 4). Furthermore, the villus height to crypt depth ratio was decreased (*P* < 0.05) in the PC compared with the NC, whereas it had a tendency to increase (*P* = 0.092) in the SYP group when compared with the PC. The number of Ki-67 positive cells in the crypt was increased (*P* < 0.05) in the PC group compared to NC, and the number of cells decreased (*P* < 0.05) in SYP when compared to the PC. The percentage of Ki-67 positive cells in the crypt was unaffected in PC when compared to NC, nor were they affected with comparing SYP to PC.

**Table 4:** Morphology of jejunum in nursery pigs fed diets supplemented with *Saccharomyces* yeast postbiotic

Item	Treatment^1^				*P* value	
	NC	PC	SYP	SEM	NC versus PC	PC versus SYP
Villus height, µm	486	430	448	30.43	0.041	0.489
Crypt depth, µm	173	187	172	12.71	0.262	0.213
VH:CD^2^	2.86	2.33	2.67	0.14	0.012	0.092
Ki-67^+3^, count	53.4	72.3	50.0	2.56	< 0.001	< 0.001
Ki-67^+4^, %	55.0	57.1	55.7	1.44	0.311	0.503

^1^NC, basal diet (no supplementation); PC, NC + challenged with F18^+^ *E. coli*; SYP, NC + *Saccharomyces* yeast postbiotics (175 g/ton of feed) + challenged with F18^+^ *E. coli*

^2^Villus height to crypt depth ratio

^3^Number of Ki-67 positive cells in the crypt

^4^Number of Ki-67 positive cells to total cells in the crypt as a percentage

### Fecal score and growth performance

**Fig. 5:**
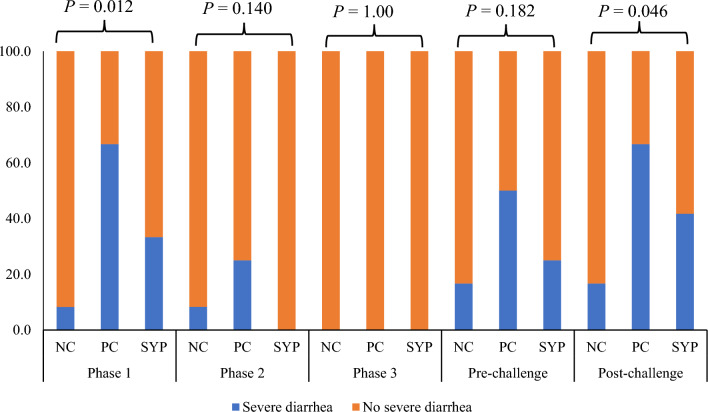
Incidence of severe diarrhea across treatments in nursery pigs. Severe diarrhea was considered to be a fecal score of 5 for 2 or more consecutive days. NC, basal diet (no supplementation); PC, NC + challenged with F18^+^ *E. coli*; SYP, NC + *Saccharomyces* yeast postbiotics (175 g/ton of feed) + challenged with F18^+^ *E. coli*

Pigs in the PC group had an increased (*P* < 0.05) fecal score on d 7 when compared with the NC, whereas the fecal score of pigs in the SYP group was decreased (*P* < 0.05) on d 7 when compared with the PC (Table 5). The PC group had an increased fecal score (*P* < 0.05) on d 12 when compared with the NC, whereas the fecal score of the SYP group was decreased (*P* < 0.05) when compared with the PC on d 12. Interestingly, the fecal score of pigs in the PC group tended to be increased (*P* = 0.087) in the PC group when compared with the NC on d 13, however, the fecal score in the SYP group was decreased (*P* < 0.05) on d 13 when compared to the SYP. Notably, the pigs in the PC group had a higher incidence of severe diarrhea (66.7%) when compared to pigs in the SYP group (41.7%), in the overall post-challenge period (Fig. 5).

**Table 5:** Fecal score of nursery pigs fed diets supplemented with *Saccharomyces* yeast postbiotic

Item	Treatment^a^				*P* value	
	NC	PC	SYP	SEM	NC versus PC	PC versus SYP
d 3–7 (Pre-challenge)	3.9	3.9	3.7	0.1	0.919	0.172
d 7 (Post-challenge)	4.0	4.7	4.1	0.2	0.010	0.031
d 8	3.8	4.5	4.1	0.2	0.011	0.123
d 9	3.7	4.1	4.0	0.2	0.131	0.732
d 10	3.8	4.4	4.1	0.2	0.059	0.454
d 11	3.9	4.2	3.9	0.2	0.217	0.191
d 12	3.3	3.8	3.3	0.2	0.043	0.029
d 13	3.5	3.9	3.2	0.2	0.087	0.003
d 14	3.6	3.5	3.5	0.2	0.764	0.868
d 15	3.4	3.4	3.2	0.1	1.000	0.260
d 16	3.1	3.3	3.1	0.1	0.448	0.313
d 17	3.1	3.2	3.0	0.1	0.717	0.570
d 19	3.1	3.0	3.0	0.1	0.611	1.000
d 21	3.0	3.1	3.0	0.1	0.732	0.732
d 23	3.1	3.0	3.0	0.1	0.144	1.000
d 25	3.0	3.0	3.0	0.04	0.168	1.000
d 27	3.1	3.0	3.0	0.03	0.067	0.915

^a^NC, basal diet (no supplementation); PC, NC + challenged with F18^+^ *E. coli*; SYP, NC + *Saccharomyces* yeast postbiotics (175 g/ton of feed) + challenged with F18^+^ *E. coli*

Body weight for pigs in the PC group was decreased (*P* < 0.05) by the end of the study when compared with the NC, however, the BW of the SYP group showed no difference when compared to the PC (Table 6). Average daily gain was decreased (*P* < 0.05) in the post-challenge period for pigs in the PC as compared with the NC, whereas there was no difference in the ADG of pigs in the SYP group compared to the PC. Average daily feed intake was decreased (*P* < 0.05) in pigs in the PC when compared to the NC, whereas SYP was unaffected when compared to the PC.

**Table 6:** Growth performance of nursery pigs fed diets supplemented with *Saccharomyces* yeast postbiotic

Item	Treatment^a^				*P* value	
	NC	PC	SYP	SEM	NC versus PC	PC versus SYP
*BW, kg*						
d 0	6.43	6.45	6.43	0.33	0.859	0.831
d 7	6.29	6.31	6.44	0.29	0.930	0.503
d 11	7.08	6.77	7.10	0.26	0.286	0.253
d 18	10.24	9.14	9.84	0.47	0.036	0.169
d 21	11.80	10.54	11.08	0.56	0.039	0.361
d 28	15.60	13.78	14.64	1.04	0.037	0.312
*ADG, g/d*						
d 0–7 (Pre-challenge)	− 19	− 19	2	15	0.999	0.315
d 7–28 (Post-challenge)	443	356	391	37	0.018	0.327
d 0–11 (P1)	59	29	62	18	0.218	0.184
d 11–21 (P2)	472	378	398	33	0.015	0.583
d 21–28 (P3)	543	463	509	73	0.112	0.356
d 0–28 (Overall)	328	262	294	27	0.032	0.290
*ADFI, g/d*						
d 0–7 (Pre-challenge)	61	63	62	10	0.860	0.952
d 7–28 (Post-challenge)	707	554	613	64	0.003	0.233
d 0–11 (P1)	117	96	106	14	0.245	0.594
d 11–21 (P2)	629	484	552	44	0.001	0.097
d 21–28 (P3)	1,100	882	946	143	0.024	0.496
d 0–28 (Overall)	546	431	475	49	0.006	0.261
*G:F*						
d 0–7 (Pre-challenge)	− 0.32	− 0.30	0.07	0.24	0.949	0.296
d 7–28 (Post-challenge)	0.63	0.64	0.64	0.03	0.731	0.752
d 0–11 (P1)	0.49	0.27	0.60	0.22	0.313	0.134
d 11–21 (P2)	0.75	0.78	0.73	0.04	0.450	0.255
d 21–28 (P3)	0.50	0.52	0.55	0.03	0.594	0.403
d 0–28 (Overall)	0.60	0.59	0.62	0.03	0.866	0.442

^a^NC, basal diet (no supplementation); PC, NC + challenged with F18^+^ *E. coli*; SYP, NC + *Saccharomyces* yeast postbiotics (175 g/ton of feed) + challenged with F18^+^ *E. coli*

## Discussion

The interactions between the host intestine and the mucosa-associated microbiota are pivotal in maintaining proper digestive functions and modulating host immune responses. The objective of this study was to develop a comprehensive view of the impacts of infection with an enteric pathogen on the mucosa-associated microbiota and intestinal immune response in nursery pigs, providing a deeper understanding of the impacts of ETEC on the intestinal health of young humans and mammals. F18^+^ *E. coli* has been identified as one of the primary strains contributing to enteric disease and post-weaning diarrhea globally for pigs [9, 37–39], and therefore, was utilized as a model for ETEC infection in this study.

Enterotoxigenic *E. coli* is characterized by its ability to produce enterotoxins. Enterotoxins are produced following colonization, and their release of triggers a cascade of effects, ultimately causing diarrhea. Enterotoxins released by ETEC can be classified as heat-labile toxins (LT) or heat-stable toxins (ST), with ST being further divided into STa and STb [40]. Both LT and ST can induce diarrhea, although their mechanisms of action differ. Heat-liable toxins activate adenylate cyclase by increasing cAMP within the enterocyte, triggering the release of chloride ions and water into the lumen, leading to watery diarrhea [41, 42]. In contrast, STa can interact with the extracellular portion of guanylyl cyclase C (GC-C) along the apical side of intestinal epithelium [43]. This interaction triggers a cascade of intracellular signaling, ultimately elevating cGMP and the subsequent activation of cGMP-dependent protein kinase, which phosphorylates the cystic fibrosis transmembrane regulator (CFTR) [44]. The CFTR is a chloride channel found on the apical side of the enterocyte, and intestinal fluid secretion is coupled with the active transport of chloride ions from the basolateral to apical surface of the enterocyte. This action of electrolyte transport can cause an increased secretion of water into the lumen, thereby increasing watery diarrhea [44]. Similarly, STb binds to microvilli in the duodenum and jejunum leading to an uptake of Ca^2+^ into the cells inducing secretion of water and electrolytes [45]. The accumulation of Na^+^ and Cl^−^ in the luminal environment stimulates the secretion of HCO3^−^, increasing the osmotic pressure, forcing more water out of the enterocytes and inducing diarrhea [46, 47]. Furthermore, STb can increase intestinal permeability by negatively affecting the tight junctions due to both the increase in intracellular Ca^2+^ and a decrease of gene expression for tight junction proteins [48].

The electrolyte imbalance associated with enterotoxins of ETEC can alter the intestinal environment and thereby influence the mucosa-associated microbiota in the jejunum [10]. The microbiota of the lumen and the mucosa differ significantly [49], and the mucosa-associated microbiota has a direct interaction with the intestinal immune cells of the small intestine [50]. As the small intestine is the major site for digestion and absorption of dietary nutrients, the interactions between the diet, potential pathogens, and the mucosal microbiota and immune system can be meaningfully investigated in the jejunum of nursery pigs [2, 3]. The effects of the disrupted electrolyte balance and increased intestinal permeability during an ETEC infection can increase incidence of diarrhea and affect the mucosa-associated microbiota [4, 51, 52]. In the present study, the adverse effects of infection with F18^+^ *E. coli* on the mucosa-associated microbiota could be observed through a shift in the populations of gram-positive and gram-negative bacteria. The LEfSe plot indicates a significant difference in the relative abundance of certain bacterial taxa, namely, *Corynebacterium, Solobacterium,* and *Bacillus,* all gram-positive bacteria, being decreased in the PC when compared with the NC. The relative abundance of these gram-positive bacteria being reduced in the PC could be due to gram-negative bacteria being better adapted to low osmolarity environments, such as those induced by ETEC [53]. The mucosa-associated microbiota has a direct interaction with intestinal immune cells through the pattern recognition receptors and this interaction can affect the host positively and negatively. Positive interactions can enhance the immune system, and negative interactions can trigger the release of inflammatory cytokines and production of oxidative stress products, that although necessary for normal immune function, excessive release of inflammatory cytokines can result in intestinal inflammation [14, 54, 55]. In the present study, a significant increase in TLR4 was observed in the PC when compared with the NC, and this could be attributed to the role of TLR4 in functioning in sensing the lipopolysaccharide (LPS) layer of gram-negative bacteria [56]. In the presence of LPS, TLR4 will initiate an intracellular signaling cascade that can induce the production of proinflammatory cytokines, however, there was a tendency for IL-6 to be decreased in the NC, which could be attributed to the unclear role of IL-6 in inflammatory responses induced by LPS. For instance, in mice, it was observed that IL-6 could perform anti-inflammatory functions [57], while another study observed that IL-6 may play a nonessential role in the induction of an inflammatory response [58].

Regarding intestinal morphology, the effects of infection with F18^+^ *E. coli* could clearly be seen through a decreased villus height and VH:CD, that is consistent with the results of recent studies utilizing an F18^+^ *E. coli* model in pigs, as reviewed by Duarte et al. [47]. Additionally, there was a tendency for the gene expression of occludin, a tight junction protein, to be increased, and there was an increased number of Ki-67 positive cells in the crypt in the PC compared to the NC. The observed increase in these parameters could be a response to intestinal damage sustained throughout the infection period, as an increase in expression of tight junction proteins generally indicates an attempt at repair of the intestinal barrier [59], whereas the increase of Ki-67 positive cells could be indicative of increased proliferation of enterocytes, another attempt at intestinal repair [60]. The aforementioned impacts on the mucosa-associated microbiota, gene expression of microbial receptors and tight junction proteins, increased fecal score and incidence of diarrhea, and decreased growth performance observed in challenged pigs can be indicative of the effects of the enterotoxins secreted by ETEC.

The dietary inclusion of postbiotics could encourage the proliferation of beneficial populations within the mucosa-associated microbiota, reducing the negative impacts associated with disruption resulting from weaning stress or pathogenic infection. Although not as commonly utilized in human applications, yeast-based products, such as yeast culture, are commonly utilized postbiotics in livestock production and have exhibited beneficial effects to animals under a variety of challenge conditions, including during suckling [61, 62] weaning [24], mycotoxin contamination [63–66], or exposure to an enteric pathogen [25, 67]. Considering the function of the intestinal immune system hinges on appropriate interactions between the intestine and the mucosa-associated microbiota, positive modulation of microbial populations of the mucosa could provide benefits in mitigating the negative effects of infection with ETEC.

Considering the known negative effects of infection with ETEC coupled with the results observed in this study, use of a *Saccharomyces* yeast postbiotic to reduce the impact of F18^+^ *E. coli* on the mucosa-associated microbiota in the jejunum could prove useful in preventing excessive damage to the small intestine. In the present study, the Chao1 α-diversity was decreased in the SYP group when compared with the PC, however, there were no differences in beta diversity between the groups. The SYP was able to make positive changes to the relative abundance of the mucosa-associated microbiota, particularly through a decreased relative abundance of gram-negative bacteria, including *Chlamydia, Prevotella, Anaerovibrio,* and *Selenomonas,* and increasing the relative abundance of gram-positive bacteria like *Sharpea* and *Bifidobacterium,* when compared to the NC. The expression of TLR4 was decreased in the SYP group when compared with the PC group, which could be associated with the decreased proportion of gram-negative bacteria present in the mucosa-associated microbiota in the SYP group, that could reduce the expression of the TLR4 receptor. The amount of protein carbonyl was decreased in the SYP when compared with the PC, indicating a decreased production of oxidative stress products. Interestingly, mTOR expression was increased in the SYP group compared with the PC, which could suggest that *Saccharomyces* yeast cell components could contain metabolites capable of activating mTOR, which plays a role in protein turnover and maintenance [68, 69] as well as enterocyte proliferation [70]. An in vitro study determined that the use of the same *Saccharomyces* yeast postbiotic utilized in this study could increase satellite cell differentiation and growth through the mTOR pathway, in muscle cells, and a similar mechanism of action could be applied to the intestinal cells [71]. Despite this, a decrease in Ki-67 positive cell count in the crypt was observed, potentially due to the pigs being past the intestinal recovery phase. Finally, the observed decrease in incidence of diarrhea in the SYP group when compared with the PC group indicates that a *Saccharomyces* yeast postbiotic could be effective in mitigating some of the negative effects associated with infection with F18^+^ *E. coli.*

## Conclusions

Challenge with F18^+^ *E. coli* increased fecal score and incidence of diarrhea, disrupted the microbiota composition in the jejunal mucosa, increased gene expression of TLR4, and had a negative effect on the jejunal morphology, negatively affecting growth performance. The addition of *Saccharomyces* yeast postbiotics could reduce the negative effects associated with F18^+^ *E. coli* infection by decreasing the relative abundance of gram-negative bacteria, thereby decreasing the gene expression of TLR4, increasing the gene expression of mTOR without elevating the secretion of proinflammatory cytokines in the jejunal mucosa, and enhancing absorptive function as evidenced by improvements in jejunal morphology, most notably a reduction in Ki-67 positive cells in the crypt. Further studies would be warranted to fully understand the mechanisms by which *Saccharomyces* yeast postbiotics mitigate the harmful effects of infection with an enteric pathogen, more specifically the unique role in activating mTOR, ultimately providing a better understanding of its role in immune system function and intestinal repair.

## Materials and methods

### Experimental design, animals, diets, and inoculation

This experiment was conducted at the Metabolism Education Unit (Raleigh, NC). Thirty-six pigs (PIC 337 × Camborough 22; 18 barrows and 18 gilts) at 6.42 ± 0.33 kg body weight (BW) were weaned at d 21 of age and were allotted to 3 treatments utilizing a randomized complete block design with initial BW blocks (heavier and lighter) and sex serving as blocks. Pigs were housed individually in pens with free access to feed and water. There were 12 replicates per treatment group. The treatment groups included a negative control (NC: basal diet, no challenge), a positive control (PC: basal diet, challenged with F18^+^ *E. coli*), and SYP (basal diet with *Saccharomyces* yeast postbiotics at 175 g/ton of feed, challenged with F18^+^ *E. coli*). Basal diets were formulated to meet NRC [72] requirements with *Saccharomyces* yeast postbiotics replacing corn in the basal diet. *Saccharomyces* yeast postbiotics were obtained from Puretein Bioscience LLC (Minneapolis, MN, USA) and are available as a commercial item. For the SYP diets, the *Saccharomyces* yeast postbiotic was mixed directly into the basal diets, replacing corn. Pigs received their respective treatments for 28 d in 3 phases: phase 1 for 11 d (d 0 to d 11), phase 2 for 10 d (d 11 to d 21), and phase 3 for 7 d (d 21 to d 28). Compositions of the experimental diets from all phases are shown in Table 7. Experimental diets were produced at the Feed Mill Educational Unit (Raleigh, NC) at North Carolina State University and a sample from each experimental diet was sent to the North Carolina Department of Agriculture and Consumer Services for proximate analysis of nutrient composition.

**Table 7:** Composition of basal diets (as-fed basis)

Item	Phase 1	Phase 2	Phase 3
*Ingredient, %*			
Corn, yellow	41.96	49.52	60.57
Whey permeate	19.00	13.00	6.00
Soybean meal, 48% CP	18.50	23.50	28.50
Poultry meal	9.00	5.00	0.00
Fish meal	5.00	3.00	0.00
HP300	3.00	1.50	0.00
Poultry fat	1.10	1.70	1.42
L-Lys HCl	0.58	0.47	0.46
L-Met	0.27	0.19	0.16
L-Thr	0.20	0.14	0.14
L-Trp	0.03	0.01	0.00
L-Val	0.02	0.01	0.03
Dicalcium phosphate	0.00	0.38	0.95
Limestone	0.44	0.68	0.87
Vitamin premix^a^	0.03	0.03	0.03
Mineral premix^b^	0.15	0.15	0.15
Salt	0.22	0.22	0.22
*Calculated composition*			
Dry matter, %	90.8	90.3	89.5
ME, kcal/kg	3,401	3,400	3,351
CP, %	24.4	22.3	19.5
SID^c^ Lys, %	1.50	1.35	1.23
SID Met + Cys, %	0.82	0.74	0.68
SID Trp, %	0.25	0.22	0.20
SID Thr, %	0.88	0.79	0.73
SID Val, %	0.95	0.87	0.78
Ca, %	0.85	0.80	0.70
STTD^d^ P, %	0.45	0.40	0.33
Total P, %	0.70	0.64	0.58

^a^The vitamin premix provided the following per kilogram of complete diet: 6,613.8 IU of vitamin A as vitamin A acetate, 992.0 IU of vitamin D3, 19.8 IU of vitamin E, 2.64 mg of vitamin K as menadione sodium bisulfate, 0.03 mg of vitamin B12, 4.63 mg of riboflavin, 18.52 mg of D-pantothenic acid as calcium pantothenate, 24.96 mg of niacin, and 0.07 mg of biotin

^b^The trace mineral premix provided the following per kilogram of complete diet: 4.0 mg of Mn as manganous oxide, 165 mg of Fe as ferrous sulfate, 165 mg of Zn as zinc sulfate, 16.5 mg of Cu as copper sulfate, 0.30 mg of I as ethylenediamine di-hydroiodide, and 0.30 mg of Se as sodium selenite

^c^SID, standardized ileal digestible

^d^STTD P, standardized total tract digestible phosphorus

Beginning on d 7, pigs in PC and SYP (challenged groups) were orally inoculated with F18^+^ *E. coli* in 4 separate doses with the following dosage concentrations: dose 1 (4.5 × 10^9^ CFU), dose 2 (6.5 × 10^9^ CFU), dose 3 (3.75 × 10^9^ CFU), and dose 4 (4.75 × 10^9^ CFU), for a final concentration of 2.0 × 10^10^ CFU. Dosage concentrations were determined based on previously conducted studies utilizing *E. coli* inoculations derived from the same cultures and animals of a similar genetic background [14, 54, 73]. The F18^+^ *E. coli* cultures were prepared utilizing strains able to produce heat-stable toxins A (STa) and heat-stable toxins B (STb), following the preparation protocol previously described [54, 74]. At each inoculation time point, the challenged groups received 1 mL of the *E. coli* suspended in a palatable carrier solution, whereas pigs in NC (unchallenged group) received a 1 mL dose of sterile saline orally.

### Experimental procedures and sample collection

Individual BW and feed intake were recorded with each change in phase to calculate average BW, average daily gain (ADG), average daily feed intake (ADFI), and gain:feed (G:F). Fecal score was recorded twice a day throughout the entire experimental period, based on a 1–5 scale: (1) very hard and dry feces, (2) firm stool, (3) normal stool, (4) loose stool, and (5) watery stool with no shape, as described by Weaver and Kim [75]. Fecal scores were as an average value for the day. Fecal scores in the pre-challenge period (d 3–7) were reported as an average of the entire period due to inconsistencies in how quickly piglets began to defecate upon placement.

At the end of the 28 d experimental period, all pigs were euthanized by exsanguination following penetration of a captive bolt gun to the head, followed by removal of the digestive tract for sample retrieval. A section of the mid-jejunum (3 m after the pyloric duodenal junction) was removed and rinsed with a sterile saline solution (0.9%) to remove digesta content. The section of jejunum was cut lengthwise and intestinal mucosa samples were obtained by scraping with a glass slide into Eppendorf tubes (2 mL), immediately placing samples into liquid nitrogen, and then storing the samples at − 80 °C for further analysis of mucosa-associated oxidative stress and immune parameters, as well as to evaluate relative abundance and diversity of the jejunal mucosa-associated microbiota. A segment from the mid-jejunum was removed and collected in a 5 mL tube, immediately placed into liquid nitrogen, and then stored at − 80 °C for further analysis of gene expression in jejunal tissue. Another segment of mid-jejunum tissue was removed, rinsed with a 0.9% saline solution, and collected in a 50 mL Falcon tube containing 10% buffered formaldehyde and stored to evaluate histology including villus height, crypt depth, and enterocyte proliferation rate.

### Profiling of the jejunal mucosa-associated microbiota

Mucosa samples collected from the mid-jejunum as previously described were used to evaluate the effect of a *Saccharomyces* yeast postbiotic on the microbial composition of nursery pigs challenged with F18^+^ *E. coli.* The samples were prepared according to protocol provided by and subsequently sent to Zymo Research (Irvine, CA) for 16S rRNA microbiome sequencing analysis. Using the ZymoBIOMICS-96 MagBead DNA Kit (Zymo Research), deoxyribonucleic acid (DNA) was extracted from the mucosa samples. Extracted DNA was then prepared for targeted sequencing using the Quick-16S Primer Set V3-V4 (Zymo Research) and the NGS Library Preparation Kit for further microbial analysis. The primers within the NGS Library Preparation Kit are designed by Zymo Research to provide complete coverage of the 16S gene. The products of DNA preparation were quantified using qPCR fluorescence readings and readings were pooled based on molarity. The resulting pooled data was refined using the Select-a-Size DNA Clean & Concentrator (Zymo Research), quantified using TapeStation (Agilent Technologies, Santa Clara, CA) and Qubit (Thermo Fisher Scientific Inc., Waltham, MA), and then the final library was sequenced utilizing on Illumina NextSeq 2000, with a p1 (CAT No. 20075294) reagent kit (600 cycles). A 30% PhiX spike-in was employed to perform sequencing, with a depth of sequencing of 20,000 × sample preparation. The DADA2 pipeline was used to infer unique amplicon sequences from the raw reads. Zymo Research Database, an internally curated 16S database based on the Greengenes and Silva 16S databases, was utilized as a reference to assign taxonomy, using Uclust from QUIIME v.1.9.1. The amplicon sequence variant (ASV) data were transformed to relative abundance (RA). Any ASV data with a RA of less than 0.5% and non-assigned sequences were combined as “Others.” Alpha and beta diversity were evaluated using the website program, MicrobiomeAnalyst (QC, CA, USA). The proportion of gram-positive to gram-negative bacteria was determined by removing “Others” and identifying the abundance of gram-positive and -negative bacteria from the known species. A linear discriminant analysis effect size (LEfSe) test was utilized at the genus level to identify significant differences in the microbial composition between the treatment groups. Beta diversity was evaluated and visualized using the Bray–Curtis distance and following procedures as previously described by Deng et al. [76].

### Relative mRNA expression of genes in jejunal tissue

Tissue samples taken from the mid-jejunum were weighed (50– mg) and were homogenized in 1 mL of TRIzol reagent (15-596-026, Invitrogen, Waltham, MA) using the Bead Mill 24 homogenizer (Thermo Fisher Scientific Inc.). Samples were homogenized twice at 4.5 m/s for 30 s and were placed on ice for 20 s in between cycles. After homogenization, samples were centrifuged for 10 min at 12,000 × *g* at 4 °C, as previously described [73]. Following centrifugation, the resulting supernatant was transferred to a 1.5 mL centrifuge tube containing 200 µL of chloroform (Thermo Fisher Scientific Inc.) and was then gently vortexed for 1 min. The mixed tubes were incubated at room temperature for 10 min, followed by another centrifugation for 15 min 12,000 ×* g* at 4 °C. Tubes were carefully removed from the centrifuge to prevent disruption of the aqueous phase, which was removed and placed into a tube containing 200 µL of isopropanol, and was then gently vortexed for 1 min to combine. The samples were incubated at room temperature for 10 min, followed by a final centrifugation for 15 min 12,000 × *g* at 4 °C. The resulting supernatant was carefully removed and the pellet was retained. Tubes were left open and allowed to dry within a fume hood for approximately 20 min, until all of the supernatant had dried off. The resulting RNA was examined for quality and quantity using spectrometry as described by Jang et al. [73]. Using a commercial kit (RevertAid First Strand cDNA Synthesis, Thermo Fisher Scientific Inc.), the extracted RNA was reverse transcribed into cDNA, following the manufacturer’s instructions. The CFX Connect Real-Time PCR Detection System (BioRad, Hercules, CA, USA) was utilized to conduct quantitative real-time polymerase chain reaction (RT-qPCR) in combination with the use of Maxima SYBR Green/ROX qPCR Master Mix (Thermo Fisher Scientific Inc.) and oligonucleotide primers synthesized by Millipore Sigma (Burlington, MA). The primers are described in Table 8.

**Table 8:** Sequence of primers for immune response and barrier function in the jejunum of pigs fed diets supplemented with *Saccharomyces* yeast postbiotics

Gene	Primer	Sequence	Accession number	Size
GAPDH	Forward	TCGGAGTGAACGGATTTGGC	NM_001206359.1	20
	Reverse	TGCCGTGGGGTGGAATCATAC		
Toll-like receptor 2	Forward	GGGCTGCGTTCATTCATCAG	XM_005653576.3	132
	Reverse	CTGCAGAGGATGGATGGCAA		
Toll-like receptor 4	Forward	CGTGCAGGTGGTTCCTAACA	NM_001113039.2	326
	Reverse	GGTTTGTCTCAACGGCAACC		
NOD^a^1	Forward	AACACCGATCCAGTGAGCAG	NM_001114277.1	230
	Reverse	AAATGGTCTCGCCCTCCTTG		
NOD2	Forward	GTGCCTCCCCTCTAGACTCA	NM_001105295.1	191
	Reverse	ACGAACCAGGAAGCCAAGAG		
mTOR^b^	Forward	TCTCTATCAAGTTGCTGGCCG	XM_003127584.6	135
	Reverse	CTAGCGCTGCCTTTCGAGAT		
Claudin-1	Forward	AAACCGTGTGGGAACAACCA	NM_001244539.1	196
	Reverse	CACATGAAAATGGCTTCCCTC		
Occludin	Forward	TCAGGTGCACCCTCCAGATT	XP_005672579.1	169
	Reverse	AGGAGGTGGACTTTCAAGAGG		
Zonula occludens-1	Forward	CAGAGACCAAGAGCCGTCC	XM_003480423.4	105
	Reverse	TGCTTCAAGACATGGTTGGC		

Glyceraldehyde 3-phosphate dehydrogenase (GAPDH) was utilized as the housekeeping gene

^a^NOD: Nucleotide binding oligomerization domain containing

^b^mTOR: Mammalian target of rapamycin

### Inflammatory cytokines, immunoglobulins, and oxidative damage products

Scraped mucosa samples taken from the mid-jejunum were weighed (500 mg) and suspended in 1 mL of PBS and stored on ice, then homogenized using a tissue homogenizer (Tissuemiser; Thermo Fisher Scientific Inc.). Homogenized samples were then transferred to a new 2 mL microcentrifuge tube to undergo centrifugation at 14,000 × *g* for 15 min, as described by Holanda and Kim [77]. Following centrifugation, the supernatant was removed, pipetted into 5 aliquots, and stored at -80 °C for further analysis.

The concentration of total protein, malondialdehyde (MDA), protein carbonyl, immunoglobulin G (IgG), immunoglobulin A (IgA), tumor necrosis factor alpha (TNF-α), interleukin 6 (IL-6), and interleukin 8 (IL-8) were measured using commercial kits following the instruction manuals. The optical density (OD) value was obtained using a plate reader (Synergy HT, BioTek Instruments, Winooski, VT) and corresponding software (Gen5 Data Analysis Software, BioTek Instruments). The concentrations for each respective product were calculated by measuring the resulting OD values against the absorbance of the standard curves following the instruction manual.

The supernatant from the aforementioned homogenization of mucosal samples was diluted (1:60) in PBS to obtain an appropriate range (20–2000 μg/mL) before measuring the total protein concentration using the Pierce BCA Protein Assay Kit (#23,225, Thermo Fisher Scientific Inc.) as previously described by Holanda et al. [78]. The absorbance was read at 562 nm and the resulting concentration of total protein for each sample was used to normalize the concentrations of subsequent measurements in each respective colorimetric assay. Using the OxiSelect TBARS MDA Quantitation Assay Kit (#STA-330, Cell Biolabs, Inc., San Diego, CA), the concentration of MDA in mucosa was measured following the procedure described by Moita et al. [79]. The working range for the standard is between 0.98 and 125 µM/L and the absorbance was read at a wavelength of 532 nm. The MDA concentration was calculated utilizing the standard and expressed as nmol/mg protein. Using the OxiSelect Protein Carbonyl ELISA Kit (#STA-310, Cell Biolabs, Inc.), protein carbonyl was measured following the procedure described Moita et al. [79]. The supernatants were diluted using PBS to achieve the protein concentration of 10 µg/mL prior to measurement. The standard was prepared within the working range from 0.375 to 7.5 nmol/mg protein. All procedures were conducted following the instruction manual. The absorbance was read at a wavelength of 450 nm and the concentration was expressed as nmol/mg protein. The concentration of IgG and IgA was measured by using the Using the ELISA kits (E101-104 and E101-102, Bethyl Laboratories, Inc., Montgomery, TX), IgG and IgA were measured as described by Holanda et al. [78]. The supernatant from the mucosal samples were diluted with PBS to 1:1600 and 1:400 for IgG and IgA, respectively, to achieve the appropriate working range for measurement. The absorbance was read at a wavelength of 450 nm and the concentration was described as µg/mg of protein. Using the Porcine TNF-α Immunoassay Kit (#PTA00, R&D Systems, Minneapolis, MN, USA), the TNF-α concentration was measured as previously described [80]. The absorbance was read at a wavelength of 450 nm and corrected at 570 nm. The final concentration of TNF-α was expressed as pg/mg protein. Using the Porcine IL-6 DuoSet ELISA kit (#DY686, R&D Systems) following Deng et al. [76]. Using the Porcine IL-8/CXCL8 DuoSet ELISA kit (#DY535, R&D Systems), the concentration IL-8 was measured as previously described [81]. The samples were diluted with reagent dilute to achieve a 1:32 ratio prior to analysis. The absorbance was read at a wavelength of 450 nm and corrected at 570 nm. All procedures were conducted following the protocol provided by the manufacturer.

### Intestinal morphology and Ki-67 in crypt cells

**Fig. 6:**
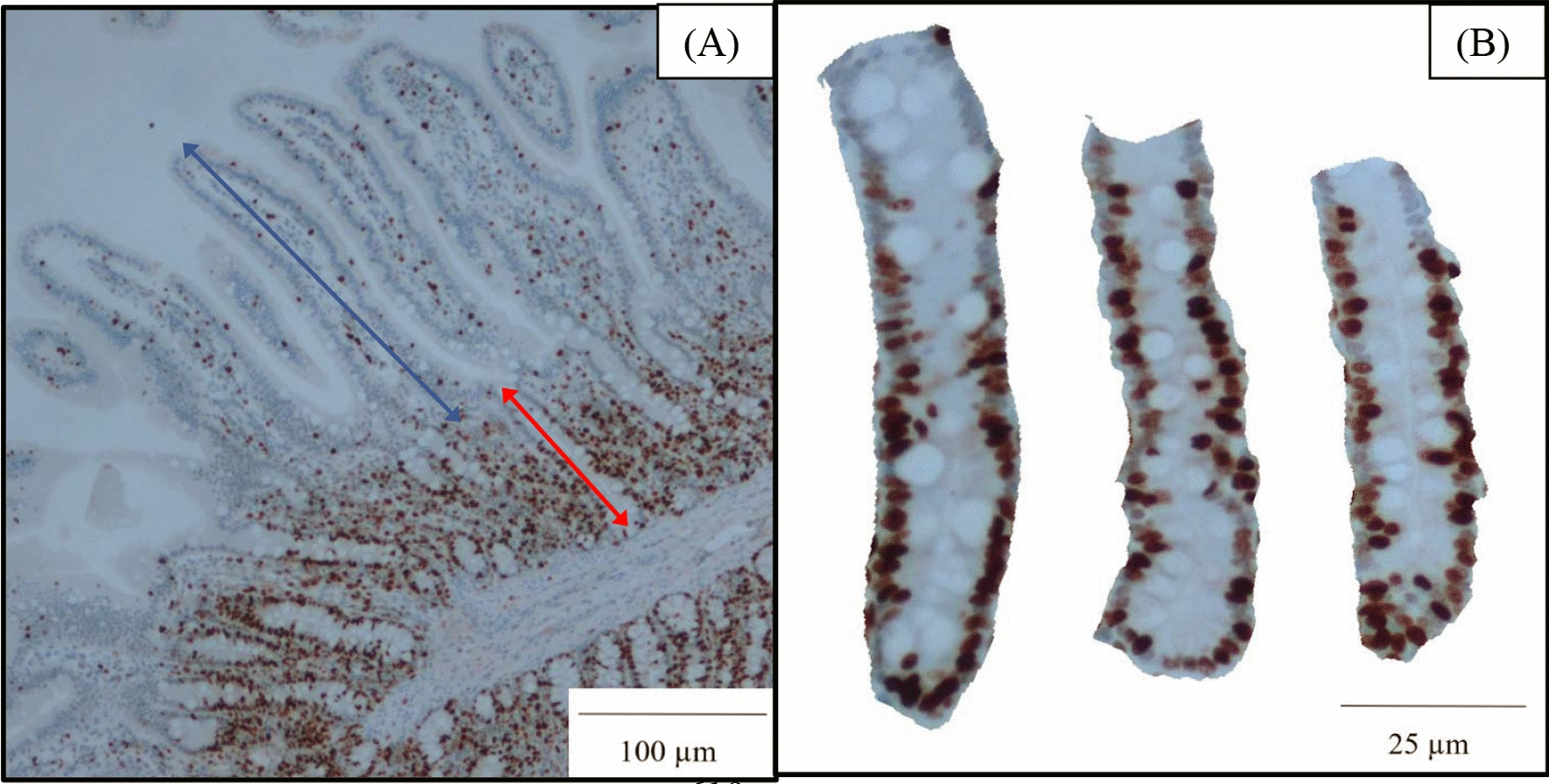
Representative images for determination of intestinal morphology and crypt cell proliferation were taken from mounted slides after immunohistochemistry (Ki67) staining. Ten images at 40 × magnification of clearly visible and well-oriented villi and their associated crypts (**A**) were obtained for each sample to measure villus height (from the top to the base of the villus, as indicated with the double arrow blue line) and crypt depth (from the base of the villus to the bottom of the crypt, as indicated with a double arrow red line). Ten images at 100 × magnification of the crypts (**B**) were captured for each sample for counting of the Ki67 positive cells as an indicator of crypt cell proliferation

Upon sampling, sections taken from the mid-jejunum of each pig were fixed in 10% formalin for 3 d. The samples were then cut transversely into two sections, placed into a cassette and transferred to a 70% ethanol solution. The sample sections were sent to the University of North Carolina School of Medicine Lineberger Comprehensive Cancer Center (Chapel Hill, NC) for dehydration, embedment, staining and immunohistochemistry of Ki-67 proteins using their internal protocol as described by Baker et al. [82]. Samples were evaluated using a microscope Olympus CX31 (Lumenera Corporation, Ottowa, Canada) and the Infinity 2–2 digital CCD software. For each sample, images of 10 intact villi and the associated crypts were captured and measured as previously described [80, 83]. The villus length was measured from the top of the villus to the junction of villus and crypt; the villus width was measured in the middle of the villus; the crypt depth was measured from the junction of villus and crypt to the bottom of the crypt. The villus height to crypt depth (VH:CD) ratio was calculated by dividing measured villus height by crypt depth. The 10 images for each sample were imported into the Teledyne Lumenera infinity analyze 7 software and Ki-67 positive cells were counted to report the number of Ki-67 positive cells in the crypt. These same images were cropped and the ImageJS software was used to calculate the percentage of Ki-67 positive cells from total cells in the crypt. Image collection and analysis of the intestinal morphology samples were executed by the same person, and 10 measurements per pig were averaged and reported as a single value per pig (Fig. 6).

### Statistical analysis

Data were analyzed utilizing the MIXED procedure in SAS 9.4 (SAS Inc., Cary, NC, USA). The dietary treatment, the main effect, was considered fixed effects, whereas, the initial BW and sex blocks were considered random effects. The individually housed pig served as the experimental unit for all measures. The data related to diarrhea incidence were analyzed using the Proc Freq of SAS 9.4. A power test was conducted to determine the appropriate number of replications needed for the study to determine statistical significance for an expected mean difference of 10–11% at *P* < 0.05. Based on previous studies conducted with pigs with a similar genetic background in the same research facility [76, 84], a coefficient of variation of 7.5% was utilized. The power of test (1 – beta) at 95% the power analysis indicated an 80%, the minimum number of replications for each treatment was 12 [85, 86]. A preplanned contrast was utilized to determine the differences of least squares means between NC versus PC, to test the effect of challenge with F18^+^ *E. coli*, and between PC versus SYP to test the effect of *Saccharomyces* yeast postbiotics on mitigating the effects of an *E. coli* infection. Results were considered statistically significant when the *P* value was less than 0.05 and were considered a tendency when the *P* value was between 0.05 and 0.10.

Alpha and beta diversity were evaluated using the website program, MicrobiomeAnalyst (QC, CA), to identify differences between the treatment groups. A linear discriminant analysis effect size (LEfSe) test was utilized at the genus level to identify significant differences in the microbial composition between the treatment groups, NC versus PC and PC versus SYP, respectively. The analysis of similarities (ANOSIM) was performed in order to evaluate the beta diversity of the mucosa-associated microbiota. The data were visualized using a principal coordinate analysis (PCoA) based on Bray–Curtis distance.

## Data Availability

The data and materials not presented in this manuscript are available from the corresponding author upon request.

## Abbreviations

ADFI: Average daily feed intake
ADG: Average daily gain
BW: Body weight
CD: Crypt depth
CP: Crude protein
DM: Dry matter
G:F: Gain to feed ratio
IgA: Immunoglobulin A
IgG: Immunoglobulin G
IL-8: Interleukin-8
IL-6: Interleukin-6
MDA: Malondialdehyde
ME: Metabolizable energy
mTOR: Mammalian target of rapamycin
NOD1: Nucleotide binding oligomerization domain containing 1
NOD2: Nucleotide binding oligomerization domain containing 2
PC: Protein carbonyl
TLR2: Toll-like receptor 2
TLR4: Toll-like receptor 4
TNF-α: Tumor necrosis factor alpha
VH: Villus height
